# Evaluating Machine Learning Affinity Prediction Methods on Novel Lead Optimization Data: A 15‐PGDH Case Study

**DOI:** 10.1002/cmdc.70531

**Published:** 2026-10-18

**Authors:** Alexander S. Rich, Leela S. Dodda, Daniel J. Price

**Affiliations:** ^1^ Inductive Bio Brooklyn New York USA; ^2^ Nimbus Therapeutics Cambridge Massachusetts USA

**Keywords:** binding affinity prediction, cofolding, free energy perturbation, lead optimization, machine learning

## Abstract

Recent machine learning (ML) methods for protein–ligand affinity prediction, such as Boltz‐2 and AEV‐PLIG, have been reported to approach free energy perturbation (FEP) accuracy on well‐known benchmarks. Even short of that accuracy, ML‐based potency models that fall between FEP and common docking scoring functions in accuracy and speed offer clear potential value. Here, we report a case study using recently disclosed data from four related ligand series targeting 15‐hydroxyprostaglandin dehydrogenase (15‐PGDH). We evaluate each ML method's predictive accuracy with a focus on when it could be used to influence synthetic priorities. AEV‐PLIG performed poorly across most series, and adding literature 15‐PGDH inhibitors to its training did not improve performance. Boltz‐2 performed well on series most similar to published inhibitors in its training, achieving accurate cofolding (median ligand RMSD ≤0.81 Å) and rank order. However, for a series exploring a new region of the binding pocket, Boltz‐2 sometimes generated less accurate poses and predicted all ligands in the series to be less potent than a published exemplar, despite experimental potency gains of up to 500x. These results highlight both the promise in well‐characterized chemical space and limitations in the novel regions where computational guidance is most needed.

## Graphical abstract

Machine‐learning‐based protein–ligand affinity prediction has the potential to meaningfully impact drug discovery given their speed relative to traditional physics‐based methods. This case study considers recently disclosed inhibitors of 15‐hydroxyprostaglandin dehydrogenase and highlights the fragility of the domain of applicability of two methods that report high benchmark performance, Boltz‐2 and AEV‐PLIG.

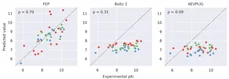

## 1 Introduction

Methods to predict small‐molecule binding affinity to a protein target are important for drug discovery, both for identifying novel chemical matter (hit finding) and for modifying known compounds to improve activity and properties (lead optimization). Free energy perturbation (FEP) is generally considered the gold standard but is computationally expensive, requiring hours of GPU time per compound. Faster scoring functions allow more chemical designs to be evaluated but have historically been insufficiently accurate to prioritize compounds for synthesis on their own. Improving the accuracy of approximate methods is a longstanding endeavor in computational chemistry, and a step change in capability, particularly as a filter to FEP, could enable more confident exploration of larger chemical modifications per synthetic cycle and accelerate dentification of a development candidate. Emerging reinforcement‐learning‐based generative models such as Reinvent [1] also evaluate thousands of ideas per generation and will benefit from potency prediction substantially faster than FEP. Many methods have been developed to meet this need, but their predictive accuracy in real‐world medicinal chemistry campaigns has generally not been well‐characterized.

Recently, machine learning methods trained on large amounts of public affinity and structural data have been reported to narrow this gap. Open‐source methods such as Boltz‐2 [2] and AEV‐PLIG [3] reportedly achieve correlation with experimental values nearly as high as FEP on common benchmarks. The closed‐source IsoDDE is claimed to exceed FEP performance [4], though methodological details have not been disclosed. The limited availability of data with both experimental structure and affinity has long been considered a fundamental challenge to robust ML‐based potency models, making these results encouraging.

## 2 Boltz‐2 and AEV‐PLIG: Reported Results and Open Questions Around Real‐World Accuracy

Boltz‐2 and AEV‐PLIG take distinctly different routes to predicting affinity. Boltz‐2 is a cofolding model, predicting the 3D structure of a protein–ligand complex from only a protein sequence and ligand SMILES. This structure feeds into a downstream affinity prediction model. AEV‐PLIG, in contrast, is a machine‐learning scoring function that predicts affinity from a provided 3D protein–ligand structure, which must be generated by another experimental or computational method.

Both leverage much larger training datasets than prior structure‐based affinity models by incorporating public affinity data that lacks experimentally determined structures. Boltz‐2 pairs affinity measurements from ChEMBL and BindingDB with self‐generated cofolded complexes, training on approximately 1.2 million measured affinity values [5, 6]. AEV‐PLIG augments the commonly used PDBBind dataset with template‐docked congeneric series drawn from ChEMBL and BindingDB, expanding from the roughly 20,000 complexes in PDBBind to around 100,000 [6, 7, 8]. These larger training sets enable both methods to achieve high performance on well‐known benchmarks. On congeneric‐series benchmarks curated for FEP methods [9], both report average per‐target Pearson r within ∼0.10 of FEP+ (Boltz‐2: 0.62 vs. 0.72; AEV‐PLIG: 0.59 vs. 0.68).

However, benchmark performance can diverge from real‐world performance for several reasons. First is the risk of information leakage from train–test similarity. Many benchmarks were not designed for ML evaluation, and even purpose‐built splits often apply insufficiently stringent exclusion criteria. Excluding complexes only when protein sequence identity exceeds 90% or ligand Tanimoto similarity exceeds 0.8, for example, is often inadequate. CDK1 and CDK2 share only 65% sequence identity yet are nearly conserved in their ATP binding sites, and a single‐atom change to a 450‐amu ligand can reduce an ECFP4 Tanimoto similarity below 0.8. One attraction of AEV‐PLIG was that its authors specifically constructed an out‐of‐distribution (OOD) test set requiring <50% protein sequence identity and <0.5 ligand Tanimoto similarity to training and reported only minor erosion of performance under this more stringent split.

But even stringent splits cannot eliminate all hidden biases. Numerous ML methods have been evaluated against the Wang et al. FEP+ benchmark [10] where potency within congeneric series in 4 of the 8 targets is strongly correlated (0.66 < *ρ *< 0.77) with heavy‐atom count (HAC), and a fifth target shows moderate correlation (ρ=0.47). This correlation makes it difficult to assess whether strong benchmark performance reflects genuine modeling of protein–ligand interactions or simply rewards ligand size. Target‐level metrics can also hide instances of Simpson's paradox [11, 12, 13]. Strong top‐line performance can mask inability to capture SAR trends within series or subseries, and conversely, good ranking within series can coincide with biases that emerge when results are pooled.

Regardless of their origin, such biases clearly exist. Multiple groups have observed that for some benchmarks, ligand‐only models perform similarly to models that embed protein–ligand interactions [14, 15]. Both Boltz‐2 and AEV‐PLIG took steps to exclude training data highly similar to their benchmark sets, but evidence suggests performance on new ligand series may still fall short of benchmark numbers. For example, when Boltz‐2 was evaluated on eight undisclosed programs at Recursion, it achieved a per‐program Pearson r of 0.39, compared to the 0.62 observed in the FEP benchmark [2].

Independent evaluation of ML models on newly released medicinal chemistry data not seen in training—and that may contain novel structure–activity relationships—is needed to inform subsequent refinement of the most promising algorithms. External evaluations of Boltz‐2 have started to emerge. Kim et al. [16] prospectively tested multiple cofolding models and showed that Boltz‐2 affinity predictions correlated with experimental values on a large set of Mac1 inhibitors. Wan et al. evaluated Boltz‐2 on large virtual screening libraries for 3CLPro and TNKS2, finding poor discrimination among top‐ranked compounds [17]. To our knowledge, there have not yet been external public evaluations of AEV‐PLIG.

We present such a case study using a recent report of 15‐hydroxyprostaglandin dehydrogenase (15‐PGDH) inhibitor optimization that spans a range of similarity to published inhibitors [18]. We treat each direction of optimization as a separate subseries, reflecting a common scope of medicinal chemistry decision‐making (representative structures in Figure 1). Series 1 systematically deconstructs the central 6,6 benzomorpholine ring system to probe ligand efficiency, and Series 2 explores scaffold hopping to 6,5 cores (e.g., the 7‐azaindole shown) and a 6,6 benzodihydropyran replacement. Series 1–3 all contain an acylpiperidine represented by some 15‐PGDH inhibitors in ChEMBL and a cryoEM cocomplex (PDB: 8CWL) [19]. Multiple 6,5 cores also appear in ChEMBL, so variations of these molecules—often sharing Murcko scaffolds—were likely in Boltz‐2's training (but not AEV‐PLIG's), based on the published training cutoff dates. Series 4 features a triazole isostere of the amide, also represented in ChEMBL but on a completely different core. Series 3 is particularly intriguing: the bromo analog shown is directly captured in ChEMBL, but analogs accessed by Suzuki coupling to displace the bromine explore a novel pocket region likely not represented in training. This provides a natural test of how prediction accuracy varies with distance from the training data.

**FIGURE 1:**
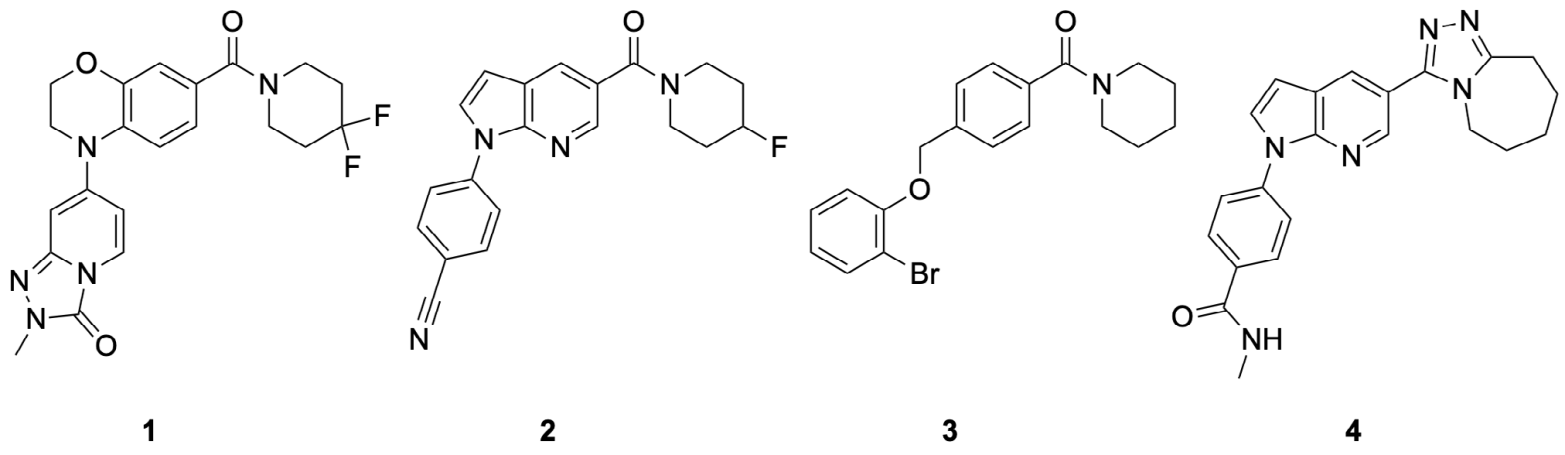
Example 15‐PGDH‐targeting compounds by series.

## 3 Methods

### 3.1 15‐PGDH Case Study

Dodda and colleagues trained a bespoke 2D ML model on initial FEP+ data and then used it to score a large virtual library for consistency with the virtual SAR [18]. The approach succeeded: 11 of 12 selected compounds (Series 3 here) were equipotent to or up to 500x more potent than the parent compound, with the 12th selected as a deliberate negative control. This success prompted us to ask whether a model trained to learn protein–ligand binding physics across diverse targets and chemistries could prioritize compounds for FEP without dedicated per‐target/series training. Using the same target and compounds therefore offered a natural case study.

Because the original campaign effectively used FEP+ [10] for potency prediction, we expect that an ML model that has learnt protein–ligand binding physics well should also be capable of predicting these experimental affinities with reasonable accuracy. In addition to Boltz‐2, we tested both the published and a custom retrained version of AEV‐PLIG, with the Vina [20] empirical scoring function as a baseline. For the noncofolding methods, ligand poses were those generated as FEP starting poses via Glide MCS (Maximum Common Substructure) constrained docking [21], followed by Vina minimization.

#### 3.1.1 Boltz‐2

Inputs to Boltz‐2 were the 15‐PGDH protein sequence, each ligand SMILES, and an NAD+ cofactor, along with a sequence alignment from the default MSA server [22]. All settings were left at default values. Affinity predictions were made from a single fixed seed. In Series 3, after observing variability in compound poses, we reran Boltz‐2 with a second seed. While this shifted some poses (see Results), affinity predictions stayed consistent with a maximal change of 0.12 log units, even among compounds where the pose changed, suggesting that seed choice has limited impact on the affinity predictions reported here.

Based on the published training cutoff date, Boltz‐2 likely saw some 15‐PGDH data. From the PDB, two structures complexed with NAD+ or NADH (2GDZ, 8FD8) and two with additional small molecules (8CVN, 8CWL) were available before the Boltz‐2 cutoff. Of the two liganded structures, 8CWL closely resembles compounds in Series 1 and 2 here, while the other is structurally unrelated though isovolumetric. One apo structure has an artificial C‐terminus that partially occludes the ligand‐binding site, potentially polluting Boltz‐2's training. Beyond these structures, ChEMBL 34 contains over 24,000 distinct compounds with recorded 15‐PGDH activity data, though all but 215 are annotated as “inconclusive.” Much of this activity data comes from an unconfirmed NCATS qHTS. The same qHTS is in PubChem with over 150,000 tested substances, including inactives excluded from ChEMBL [23].

#### 3.1.2 AEV‐PLIG

We evaluated two versions of the model, each a 10‐fold ensemble as in the original publication, averaging predictions across folds. First, we used the default AEV‐PLIG trained on PDBBind, BindingNet, and BindingDB‐DCS, none of which contain 15‐PGDH complexes. We also retrained AEV‐PLIG with 40 additional ligands and potencies from Ria et al. [24], docked into the same receptor, to assess the impact of relevant 15‐PGDH training data; these Ria compounds occupied a similar binding mode to our test series but were structurally distinct, with maximum Morgan‐2 Tanimoto similarities of 0.369 to Series 1 and 2, 0.625 to Series 3, and 0.323 to Series 4. We used the published training script (https://github.com/oxpig/AEV‐PLIG) with no changes to hyperparameters or training protocol beyond the added complexes. The retrained ensemble fit the added 15‐PGDH training data well (MAE = 0.06, Spearman ρ = 0.99), confirming the model is capable of fitting these complexes despite their small share of the training set. As a positive control, we confirmed both ensembles reproduced published AEV‐PLIG performance on the 8‐target JACS FEP+ benchmark [10], which is contained in Schrödinger's public binding free energy benchmark repository (https://github.com/schrodinger/public_binding_free_energy_benchmark). Mean per‐target Spearman ρ was within 0.01 of the value reported by the AEV‐PLIG authors (https://github.com/matthewtwarren/aev‐plig‐paper) for the default model, and within 0.03 for the retrained model.

#### 3.1.3 Vina Scoring Function

Vina predictions were generated with the GNINA command‐line tool using minimize to score the nearest local minimum [20, 25].

#### 3.1.4 Heavy Atom Count (HAC)

As a final baseline, we computed heavy‐atom count (HAC) per compound.

#### 3.1.5 Evaluation Metrics

To compare methods, we focused on the Spearman rank correlation coefficient (ρ). This allows comparison across methods on different scales and reflects a real‐world objective: ranking compounds by synthetic priority.

#### 3.1.6 Statistical Analysis

To assess whether differences in ρ between methods were statistically significant, we used a nonparametric paired bootstrap (10,000 resamples) to compute 95% confidence intervals and *p*‐values for paired comparisons. Within each series (and for the pooled dataset), comparisons of the five alternative methods against FEP+ were corrected for multiple testing via Benjamini–Hochberg FDR, applied independently per series. Given the small per‐series sample sizes (n=12–18), confidence intervals are wide, and nonsignificant results should be interpreted as limited power rather than equivalence.

## 4 Results

### 4.1 Validation of Boltz‐2 Cofolding Accuracy

Before evaluating affinity predictions, we assessed Boltz‐2's cofolding accuracy for each series. We aligned each cofolded complex to an experimental structure of 15‐PGDH bound to the first structure in Figure 1 (PDB: 9PFL). For Series 1, 2, and 4, we calculated ligand RMSD relative to the Glide MCS‐docked pose used by the other methods. This assumes the MCS‐docked pose is correct, and several lines of evidence support that assumption. First, cryoEM and crystal structures of 15‐PGDH bound to structurally distinct ligands have protein heavy‐atom RMSDs ≤0.7 Å—within experimental uncertainty—so we expect no ligand‐induced changes to the binding pocket. Second, most ligands are isovolumetric and match a single pharmacophore well represented by existing experimental costructures, giving high confidence in the MCS‐docked pose. Finally, the MCS‐docked poses are stable in FEP+ terminal window simulations.

Series 3 is more complicated. FEP+ equilibration revealed that some members of the series remain as designed, displacing several water molecules, while others move slightly to directly coordinate with those waters, not unlike some published inhibitors (e.g., the solvent‐facing pyridotriazolone of compound 1). However, this pose pushes the adjacent phenyl ring in the opposite direction, picking up additional hydrophobic interactions with Met143—also not seen in published inhibitors—while displacing experimentally observed crystallographic waters. Because equilibrated complexes were stable in simulation and FEP+ reasonably reproduced experimental potencies, we used a representative snapshot from the terminal window simulation of each member as the reference. The nuance of water coordination versus displacement for similar ligands poses an intriguing challenge for Boltz‐2.

Overall, cofolding accuracy was good across series (Table 1). For Series 1, 2, and 4, Boltz‐2 produced poses in close agreement with the docked structures, with median RMSDs of 0.55, 0.56, and 0.81 Å, respectively. This was perhaps expected given the similarity to a structure presumed to be in training. For Series 3, which was topologically and volumetrically most dissimilar from published structures, performance was decent but uneven (mean 1.61 Å, median 1.19 Å; Table 1). Visual inspection showed that Boltz‐2 correctly placed the portion unchanged from literature compounds, but variability was higher in the solvent‐exposed group. Example poses compared to explicit simulation snapshots are shown in Figure 2, ranging from high agreement (Figure 2A) to slightly shifted (Figure 2B) to a fully flipped solvent‐exposed group (Figure 2C).

**TABLE 1:** Per‐series ligand RMSD (Å) between Boltz‐2 cofolded poses and reference poses.

Series	n	Mean	Median	Std. Dev.
1	11	0.74	0.55	0.35
2	7	0.66	0.56	0.33
3^a^	13	1.61	1.19	1.04
4	12	0.86	0.81	0.25

a Series 3 RMSD were measured against FEP‐derived simulation snapshots as the MCS docked pose was not always sufficient to capture an equilibrated cocomplex. Series 3 RMSD represents the average of two repeat predictions.

Variation in Boltz‐2 positioning of the ligand prompted us to examine the repeat predictions generated with a second random seed. While most poses were stable across repeats (median RMSD 0.29 Å between poses), two compounds showed substantial deviations, with RMSDs exceeding 4 Å from the reference FEP pose. Figure 2B,C show repeat predictions of one of these compounds. The repeated occurrence of the flipped pose prompted us to ask whether it might be stable but unsampled in the relatively short (10 ns/window) relative binding free‐energy calculations starting from the other conformation. However, absolute binding free energy calculations starting from either conformation confirmed the reference FEP poses as more stable for several members (data not shown). We also observed that even when Boltz‐2's predicted pose shifted meaningfully, its affinity prediction did not; pIC50 values were within 0.12 for all pairs of repeat predictions and were within 0.05 for each of the compounds that showed meaningful pose changes. Selection among Boltz‐2 poses therefore appears to have limited impact on predicted affinities, though it of course matters for uses of the pose itself, such as structure‐based design.

### 4.2 Series 1 and 2: Core Modifications

Series 1 and 2 explored modifications to the bicyclic core, leaving both ends of the molecules unchanged relative to published inhibitors [18]. Boltz‐2 achieved a strong correlation with experimental potency (ρ=0.68), not significantly different from FEP+ (ρ=0.64; Figure 3), though power is limited at this sample size. Notably, Boltz‐2 predictions span a compressed potency range (7.3≤pIC50≤8.0), apparently reflecting the potency range of similar molecules in training: most ChEMBL molecules containing an acylpiperidine have potencies 6.8≤pIC50≤7.9. The Vina scoring function also showed good correlation (ρ=0.55). AEV‐PLIG performed poorly in both default (ρ=0.14) and retrained (ρ=0.11) versions.

**FIGURE 2:**
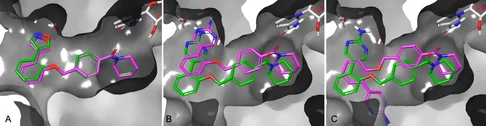
Comparison of poses of exemplar Series 3 compounds. Binding pocket, green ligand, white NAD+, and select structured water molecules are snapshots from terminal window of FEP+ simulation. Part of the enclosed binding pocket removed for clarity. Pink ligands are predictions from Boltz‐2 after alignment of the entire protein–ligand–cofactor complex. Examples showing high agreement between Boltz‐2 and FEP+ snapshot (Panel A, RMSD = 0.55 Å) and moderate agreement (Panel B, RMSD = 1.9 Å). Panel C shows repeat prediction with the same compound in Panel B, but with gross deviation in orientation.

**FIGURE 3:**
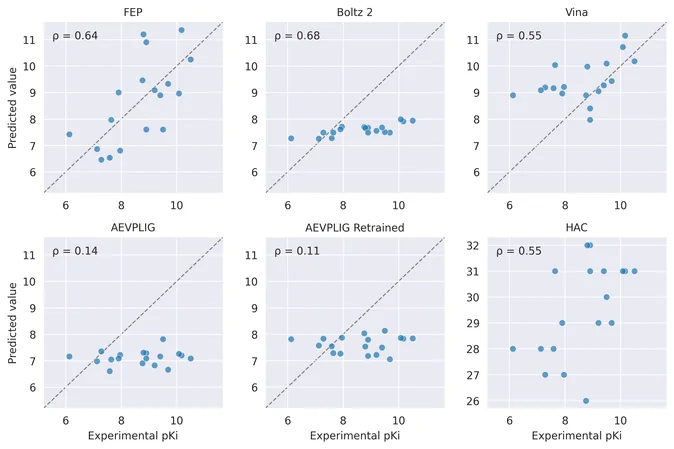
Affinity prediction results on Series 1 and 2. Each panel shows predicted values versus experimental pK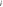 for a different scoring method. Spearman ρ is shown in each panel.

However, heavy atom count (HAC) also achieved ρ=0.55, matching Vina and approaching Boltz‐2. This correlation with molecular size raises the earlier concern that some methods’ performance may be driven by a simple bias toward larger ligands. With only 18 ligands, differences between methods are mostly not statistically significant (Table 2).

**TABLE 2:** Spearman ρ with experimental potency (95% bootstrap CI) for each scoring method and compound series. RT denotes the AEV‐PLIG model retrained with 15‐PGDH inhibitors. Asterisks denote methods whose ρ differs significantly from FEP+ after Benjamini–Hochberg correction ($p_{\mathrm{adj}}<0.05$, $p_{\mathrm{adj}}<0.01$).

Method	All series, n = 43	Series 1 & 2, n = 18	Series 3, n = 13	Series 4, n = 12
FEP+	0.70 [0.48, 0.84]	0.64 [0.24, 0.83]	0.78 [0.37, 0.96]	0.66 [0.04, 0.96]
Boltz‐2	0.31 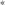 [−0.00, 0.58]	0.68 [0.22, 0.90]	0.17 [−0.52, 0.74]	0.55 [−0.00, 0.91]
Vina	0.45 [0.14, 0.68]	0.55 [0.05, 0.85]	0.57 [−0.07, 0.85]	0.67 [0.17, 0.91]
AEV‐PLIG	0.09 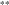 [−0.22, 0.38]	0.14 [−0.34, 0.58]	0.25 [−0.32, 0.73]	−0.14 [−0.70, 0.47]
AEV‐PLIG RT	0.06 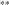 [−0.24, 0.35]	0.11 [−0.43, 0.50]	0.43 [−0.27, 0.91]	−0.44 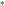 [−0.82, 0.18]
HAC	0.09 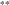 [−0.23, 0.40]	0.55 [0.17, 0.76]	−0.44 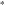 [−0.90, 0.25]	−0.15 [−0.66, 0.42]

### 4.3 Series 3: Exploring a Novel Subpocket

As described in Dodda et al. [18], 2–3 high‐energy waters were identified near existing ligands, and a series of new compounds derived from a published inhibitor (Compound 4 from Dodda et al., shown as the Series 3 example in Figure 1) were designed to displace those waters. All but one increased inhibition, though as noted above, the exact mechanism of the potency gains was ligand‐dependent. FEP correlated well with measured potencies (ρ=0.78; Figure 4), presumably capturing those ligand‐dependent interaction modes. Subtle shifts in ligand position yielding different interactions—and thus different entropic and enthalpic contributions—pose a difficult scoring challenge for ML methods, which must implicitly deduce the presence of structured water from the protein structure and generically learn which ligand–protein distances achieve potency gains via displacement or coordination.

**FIGURE 4:**
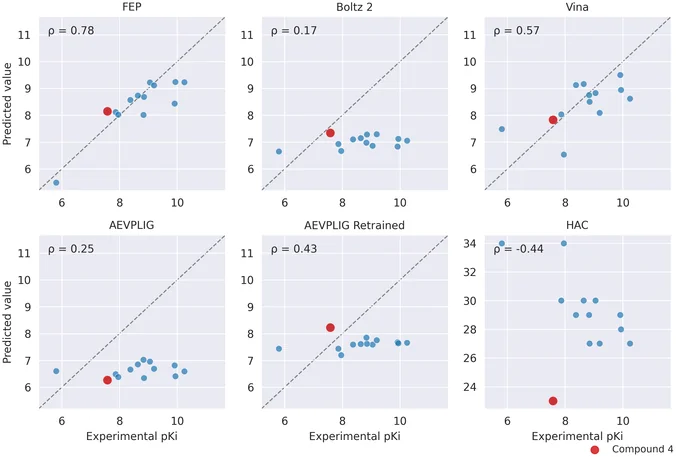
Affinity prediction results on Series 3. Each panel shows predicted values versus experimental pK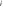 for a different scoring method. Spearman ρ is shown in each panel.

Compounding this, Boltz‐2 did not exactly reproduce the FEP+‐generated poses in all examples in this series (see Section 4.1). Regardless, Boltz‐2 did not predict any series member to be more potent than the brominated parent (Compound 4)—including those whose binding mode it predicted accurately and despite the higher HAC of the derivatives. Compound 4 was the only series member whose potency was likely in training.

The fact that compounds in this series were selected to be consistent with the topology of molecules that previously scored well in FEP means that directly comparing the number of compounds with improved activity relative to FEP has some inherent bias. However, predicting no compounds to be more potent is concerning. Furthermore, the compounds were not selected based on FEP's ability to reproduce experiment, so the Spearman coefficient relative to experiment should represent an unbiased statistic on the medicinal chemistry objective of setting synthetic priority. Ranking power with Boltz‐2 here was poor (ρ=0.17). Predicted rank does not appear related to structure‐prediction quality. For example, the most potent compound was ranked by Boltz‐2 as middling despite a 0.5 Å RMSD to the FEP‐derived pose. Boltz‐2 did correctly identify the least potent compound, whose bulk cannot easily fit the pocket without considerable contortion, suggesting some generalizable learning of sterics and/or conformational energetics.

In this series, both Vina and the two AEV‐PLIG variants score the MCS‐docked pose, which does not capture the ligand‐dependent binding mode differences. We deliberately did this to mirror a real‐world scenario where FEP/simulation would not be run upstream of scoring for pose refinement.

Despite this, Vina performed relatively well on Series 3 (ρ=0.57). Although Vina has no explicit term for high‐energy water displacement and no water was present for coordination, it did find the additional protein–ligand interactions in the new analogs favorable. Retraining AEV‐PLIG with published compounds also yielded a moderate rank correlation on this series (ρ=0.43); Series 3 is the series most similar to the added Ria compounds (maximum Morgan‐2 Tanimoto 0.625, vs. 0.369 and 0.323 for the other series). It nonetheless shared Boltz‐2's bias toward the published compound, ranking it as most potent.

Finally, in Series 3, larger HAC was associated with worse affinity, so Vina's ranking cannot be explained by a bias toward larger ligands. Differences between methods were not statistically significant given the small sample size (but see Section 4.5).

### 4.4 Series 4: Bioisosteric Replacement

Finally, Series 4 explored compounds containing a fused cycloalkyl triazole. The moiety was published to ChEMBL on a completely different scaffold as a high‐throughput screen hit with subsequent analog follow‐up. Dodda et al. predicted the triazole nitrogens to be a bioisostere of the acylpiperidine oxygen present in most published 15‐PGDH inhibitors, inspiring a series hybridizing this and related groups with pharmacophoric concepts of known potent inhibitors [18]. This series also poses an intriguing challenge for ML‐based models. The potency range of exemplars in ChEMBL (5.4≤pIC50≤7.7) does not overlap that of this series (8.0≤pIC50≤10.0), so ML models must capture additive SAR. Mixing and matching pieces across series to obtain topologically novel but pharmacophorically conservative molecules is a common medicinal chemistry strategy.

Vina (ρ=0.67) correlated well with experimental potency on this series (Figure 5), comparable to FEP+ (ρ=0.66). Boltz‐2 also showed positive correlation (ρ=0.55), again with a compressed potency range aligned with the more potent exemplars in the training distribution. AEV‐PLIG was again not predictive (ρ=−0.14 default; ρ=−0.44 retrained). HAC varied little within the series and showed no correlation with potency (ρ=−0.15), so the positive results from Boltz‐2 and Vina cannot be attributed to molecular size.

**FIGURE 5:**
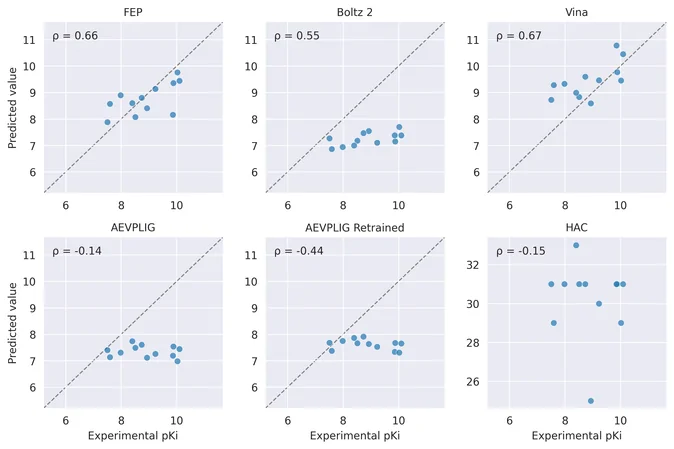
Affinity prediction results on Series 4. Each panel shows predicted values versus experimental pK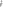 for a different scoring method. Spearman ρ is shown in each panel.

### 4.5 Pooled Analysis Across Series

The per‐series analyses evaluated each method's ability to rank compounds *within* a series involving a single change at a fixed position. However, chemists must also prioritize compounds *across* series—deciding, for example, whether to invest synthetic effort in Series 3 or Series 4 analogs or even to hybridize the two into another series. To test this cross‐series ranking ability, we pooled all 43 compounds and computed Spearman correlations with experimental potency (Figure 6).

**FIGURE 6:**
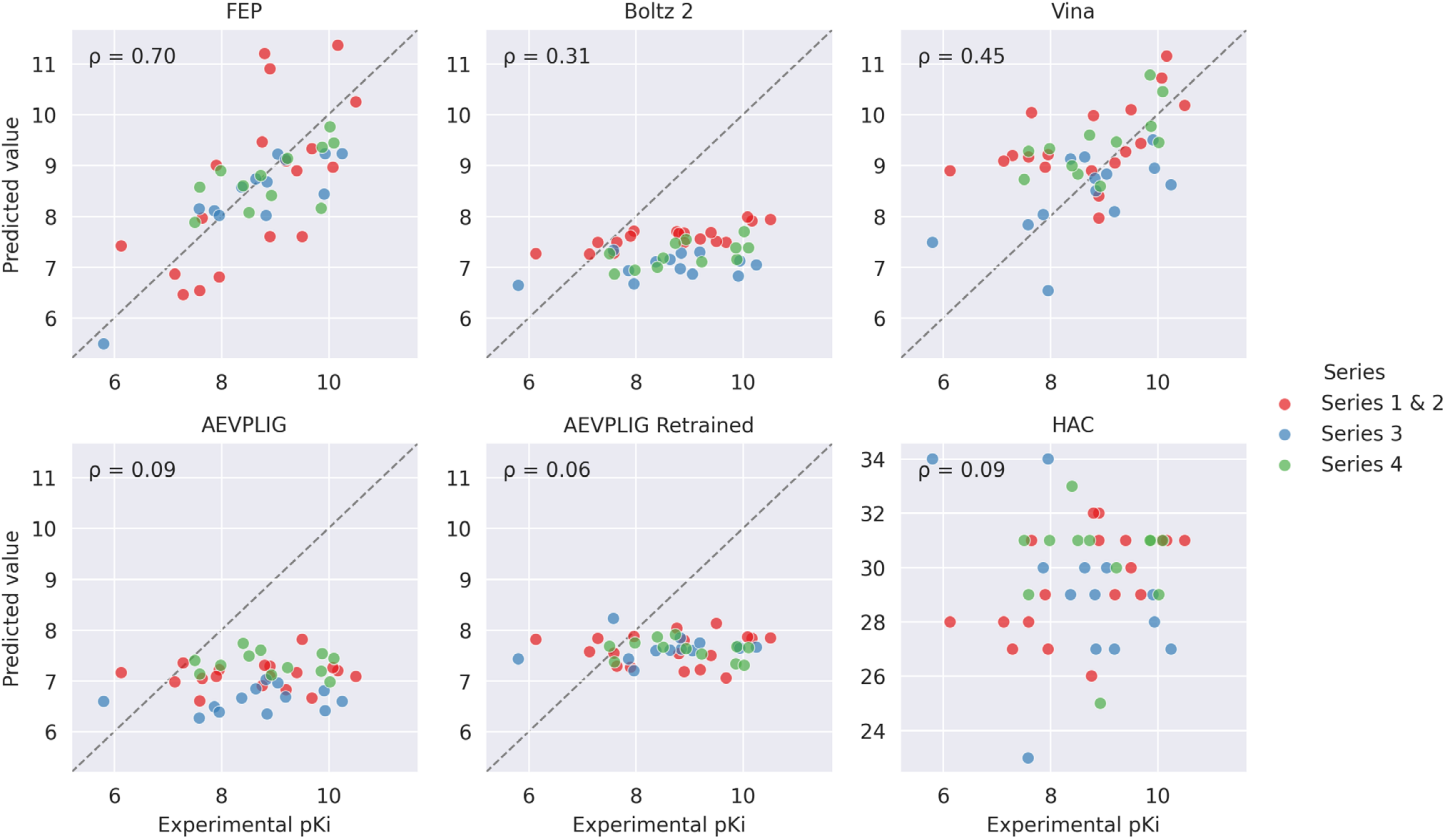
Affinity prediction results across all series. Each panel shows predicted values versus experimental pK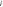 for a different scoring method, with points colored by series. Spearman ρ is shown in each panel.

Relative to the FEP+ approach used by Dodda and colleagues (ρ=0.70), Vina showed moderate pooled correlation (ρ=0.45). Boltz‐2's pooled correlation (ρ=0.31) was considerably lower than its within‐series performance on Series 1, 2, and 4, and significantly lower than FEP+ ($p_{\mathrm{adj}}<0.05$). The rank correlations of AEV‐PLIG and HAC remained near zero.

The drop in Boltz‐2's performance upon pooling reflects a Simpson's paradox‐like effect [11]: even when the model ranks compounds reasonably within series, systematic between‐series biases degrade the overall correlation. Specifically, Boltz‐2 predicted that Series 3 compounds—which experimentally are as potent as other series—would be uniformly less potent than Series 1 and 2 compounds (Figure 6, Boltz‐2 panel). A similar but less pronounced effect is visible for Series 4. These biases likely stem from Boltz‐2's training data; the physics‐based FEP+ method and the empirical Vina scoring function do not exhibit this between‐series distortion.

## 5 Conclusion

In our search for a fast, general ML‐based potency model to prioritize compounds, we evaluated Boltz‐2 and AEV‐PLIG: two recent approaches with competitive performance on well‐known benchmark datasets. Although limited to a single target and a relatively small number of molecules, this study covers commonly encountered themes in early discovery: limited structural and chemical precedence, scaffold hopping, hybridization of pharmacophoric concepts or chemical moieties across series, and exploration of new vectors. For reasons described in the introduction, large benchmarks often overestimate prospective performance on any given medicinal chemistry effort, so focused evaluations such as this can be useful to practitioners. Several trends here point toward concrete conclusions.

First, the ML scoring function AEV‐PLIG was not predictive for this target under the tested workflow. Performing worse than Vina on the same MCS‐docked poses is consistent with overfitting to specific training complexes rather than learning the underlying physics of protein–ligand binding, though the comparison is not entirely even‐handed since those poses were Vina‐minimized, and Vina is therefore scored on structures optimized under its own objective. The retrained model's near‐perfect fit to its 40 added training complexes (MAE = 0.06, Spearman ρ = 0.99) confirms it is capable of memorizing training data. However, this comparison alone cannot distinguish overfitting from other explanations such as target‐ or chemotype‐specific domain shift or pose sensitivity, and our observations are limited to a single target. The AEV‐PLIG authors built a more challenging OOD hold‐out than is typical and reported reasonable performance on it, making the discordance with our experience striking. We lack a fully satisfying explanation, but the gap highlights the value of monitoring per‐target/series performance alongside aggregate statistics since that is how any published model will ultimately be used.

Retraining with 15‐PGDH inhibitors did not consistently improve performance: rank correlation rose for Series 3 but fell for Series 4, with little change on Series 1 and 2 or in the pooled analysis. Series 3 was also the series most similar to the added compounds, consistent with any benefit being localized rather than general. It is unclear whether 40 additional complexes are simply dwarfed by the rest of the training set, additional hyperparameter optimization is needed for a new target, something learnt from the rest of the training set is counterproductive here, or some of the reported OOD performance reflects the hidden biases discussed above. The latter possibility underscores the continued need for research into hidden benchmark biases and for more rigorous ML‐focused benchmarks, such as the recently developed PLINDER [26].

Our results for Boltz‐2 are more promising but nuanced, revealing a spectrum of cofolding model performance that depends on the novelty of the binding hypothesis being explored. We first highlight that the Boltz‐1x/2 team made a significant cofolding advance by introducing steered diffusion to maintain reasonable chemical geometries, which was evident in every example tested here. For Series 1, 2, and 4—where compounds are structurally similar to published inhibitors—Boltz‐2 achieved both accurate cofolding and competitive affinity predictions while also offering the practical advantage of generating its own poses without requiring template‐based docking. Particularly in Series 4, where HAC was uncorrelated with potency, Boltz‐2's positive correlation suggests information gain over the HAC baseline and some transferable physics. Together, performance on these three series points to a clear but limited use case: prioritizing relatively conservative, isovolumetric changes at a single position.

For Series 3, however, which explored a novel subpocket through displacement and/or coordination of high‐energy, structured waters, Boltz‐2's performance degraded. The exact RMSDs here are imperfect, since Boltz‐2 predictions likely reflect ∼100 K (i.e., learned from flash‐frozen crystal and cryoEM samples) yet were compared to snapshots from room‐temperature simulations. Qualitatively, the cofolded poses generally recapitulated FEP‐derived poses in most but not all cases. Even so, the affinity predictions rewarded the published parent compound while penalizing ligands making interactions not seen in training. Used prospectively to evaluate these specific changes, none would have been synthesized. On the one hand, the inability to capture potency gains via structured water might be dismissed as too tall an order for any model not explicitly representing water. On the other, in nearly all cases where a ligand is grown into a new part of a binding pocket, water will be displaced. Furthermore, the empirical Vina scoring function captured some of the potency improvements without explicitly modeling water.

The performance in this series speaks to how precarious the domain of applicability (DoA) of ML potency models can be: even relatively small changes (e.g., adding four heavy atoms) can push predictions outside the DoA. This pattern has important implications for the practical use of this and perhaps other ML potency models in drug discovery. The cases where computational guidance is most valuable are precisely those where medicinal chemists are exploring novel binding hypotheses—new subpockets, new interaction types, or new scaffolds. Yet these are also the cases where ML models are most likely to struggle because the relevant binding modes and corresponding potencies may be poorly represented in training data. Our results suggest the DoA for accurate cofolding affinity prediction may be narrower than benchmark performance suggests, potentially limited to regions of chemical space well‐covered by existing structural and affinity databases.

Two additional concerns emerged. First, the compressed potency range in Boltz‐2's predictions appears to reflect the smaller, less‐potent distribution of experimental IC50s for similar 15‐PGDH inhibitors in training. This precludes targeting efficiency metrics as a medicinal chemistry objective and may also indicate the model struggles with SAR additivity or synergy. This warrants monitoring in future studies since mere representation in training may be insufficient—the shape of the potency distribution likely matters as well. Second, while poor performance on Series 3 surely muddies Boltz‐2's pooled analysis, there appears to be a systematic bias by series that raises concerns of Simpson's paradox. The effect was slight outside Series 3 but warrants further monitoring to define appropriate usage.

In many ways, this case study is a relatively favorable scenario for cofolding models: 15‐PGDH has structural and potency precedence, liganded structures show no significant ligand‐induced changes, and most series tested involve highly conservative modifications relative to known inhibitors. Given the fragility of the DoA observed here, performance could be suspect for completely novel targets, mechanisms of inhibition, or chemical matter. There are also cases, such as examples of allosterism, where cofolding models like Boltz‐2 fail to produce reasonable binding modes altogether [27, 28]. While evaluation on many more targets and compound series is needed to draw definitive conclusions, this case study illustrates a key challenge for ML‐based affinity prediction: these methods appear most reliable where experimental data is already abundant, but less reliable in the novel regimes where computational guidance would be most impactful.

Finally, these conclusions rest on a single case study. Additional published studies on unseen data, such as the recent Mac1 results [16], will refine our understanding of how well cofolding affinity models and ML scoring functions work. Ideally, organized cross‐institution partnerships will form to evaluate ML affinity prediction, as CACHE has for hit‐finding and OpenADMET has for ADMET [29, 30]. The recently formed OpenBind consortium is beginning to produce new datasets that could easily be used for such efforts [31]. Public, blinded benchmarks will give practitioners the knowledge to decide when and how to use new ML methods for affinity prediction.

## Conflicts of Interest

A.S.R. is an employee of Inductive Bio. L.S.D. and D.J.P. are employees of Nimbus Therapeutics.

## Data Availability

The data that support the findings of this study are openly available in inductive‐bio‐misc‐collab/ml‐affinity‐15‐PGDH‐case‐study at https://github.com/inductive‐bio‐misc‐collab/ml‐affinity‐15‐PGDH‐case‐study.
